# Jagged 2 inhibition attenuates hypoxia-induced mitochondrial damage and pulmonary hypertension through Sirtuin 1 signaling

**DOI:** 10.1371/journal.pone.0297525

**Published:** 2024-01-26

**Authors:** Hanhan Liu, Zhou Pan, Xiaofeng Wu, Cheng Gong, Junbo Hu

**Affiliations:** 1 Department of Pathology, Maternal and Child Health Hospital of Hubei Province, Tongji Medical College, Huazhong University of Science and Technology, Wuhan, China; 2 DepartmentofRespiratoryandCriticalCareMedicine, Renmin HospitalofWuhan University, Wuhan, China; 3 DepartmentofRespiratoryandCriticalCareMedicine, TaiheHospitalofShiyan, HubeiUniversityofMedicine, Shiyan, China; 4 Department of Hepatobiliary and Pancreatic Surgery, Zhongnan Hospital of Wuhan University, Wuhan, China; Xuzhou Maternity and Child Health Care Hospital affiliated to Xuzhou Medical UniversityCare Hospital Affiliated to Xuzhou Medical University, CHINA

## Abstract

Notch pathway has played a significant role in the pathophysiology of pulmonary hypertension (PH). However, the role of Jagged 2 (Jag2), one ligand of Notch, remains to be elucidated.Therefore, determining the contribution of Jag2 to PH and its impact on pulmonary artery smooth muscle cells (PASMCs) was the aim of this investigation. Adeno-associated virus-mediated Jag2 inhibition was used to explore the role of Jag2 in peripheral pulmonary vascular remodeling assessed in a rat model of chronic hypoxia (10% O_2_, 4 weeks) induced pulmonary hypertension. In vitro, the effect of Jag2 silencing on hypoxia (1% O_2_, 24h) induced rat PASMCs was determined. Group differences were assessed using a 2-sided unpaired Student’s t-test for two groups and one-way ANOVA for multiple groups. *Jag2* upregulation was first confirmed in rats with sustained hypoxia-induced PH using publicly available gene expression data, experimental PH rat models and hypoxia induced rat PASMCs. Jag2 deficiency decreased oxidative stress injury, peripheral pulmonary vascular remodeling (0.276±0.020 vs. 0.451±0.033 μm, *P*<0.001, <50μm), and right ventricular systolic pressure (36.8±3.033 vs. 51.8±4.245 mmHg, *P*<0.001) in the chronic hypoxia-induced rat model of PH. Moreover, *Jag2* knockdown decreased proliferation (1.227±0.051 vs. 1.45±0.07, *P* = 0.012), increased apoptosis (16.733%±0.724% vs. 6.56%±0.668%, *P*<0.001), and suppressed mitochondrial injury in hypoxia–treated rat PASMCs. Jag2 inhibition restored the activity of the Nrf2/HO-1 pathway, which was abolished by Sirtuin 1 deficiency. These findings show that Jag2 is essential for modulating pulmonary vascular dysfunction and accelerating PH, and that inhibition of Jag2 expression suppresses the progression and development of PH.

## Introduction

Pulmonary hypertension (PH) is a life-threatening condition characterized by elevated pulmonary arterial pressure leads to dyspnea, exercise intolerance, and eventually right heart failure[1]. Although the hemodynamic criterion of a mean pulmonary arterial pressure >3.33 kPa (25 mmHg) at rest is useful for defining PH clinically, the etiology and clinical manifestations of PH vary. Hypoxia-induced pulmonary arterial hypertension represents the third classification among the five categories of pulmonary hypertension outlined by the World Health Organization: PH associated with chronic lung diseases or hypoxemia.

Continuous exposure to hypoxia leads to pulmonary vasoconstriction and structural changes that increase vascular stiffness, reduce arterial luminal diameter, and increase blood flowresistance.Together with an increase in hematocrit and thus blood viscosity, hypoxia-induced PH increases the burden on the right ventricle[2].Endothelium-derived vasoconstrictors (such as endothelin and thromboxane) enhance hypoxic pulmonary vasoconstriction, while endothelium-derived vasodilators (such as nitric oxide and prostacyclin) inhibit it. However, the primary mechanism lies within the mitochondria of pulmonary artery smooth muscle cells (PASMCs), encompassing their unique contractile proteins and signaling molecules.[3]. PASMCs mitochondria have the ability to detect hypoxia and adapt to fluctuations in reactive oxygen species (ROS) generation by coordinating intracellular voltage- and chemo-sensitive K^+^ and Ca^2+^ channels. [4]. Several previous studies have shown that hypoxia induces ROS production in mitochondria, a decrease in mitochondrial membrane potential, and consequent mitochondrial dysfunction[5–7]. Improvementsin hypoxia-induced mitochondrial dysfunction therefore alleviate the proliferative and anti-apoptotic phenotype of PASMCs and slow the progression of experimental PH.

A growing body of research shows that Notch signaling is important for vascular smooth muscle cell proliferation and differentiation [8]. Among the ligands responsible for Notch receptor activation, Jagged 2 (Jag2) plays a prominent role alongside Jag1, Dll1, Dll3 and Dll4. These ligands, encoded by the Jag and Dll gene families, engage in direct intercellular contact (trans-activity) or autocrine contact within the same cell (cis-activity) with endothelial-bound or smooth muscle-bound ligands. The Notch receptor, a transmembrane protein encompassing Notch 1 to 4, acts as the receiver of these signals[9].The intracellular structural domain (ICD) of the Notch receptor is liberated into the cytoplasm of the signal-receiving cell following a cascade of proteolytic events triggered by ligand binding to the Notch receptor. Subsequently, the ICD translocates to the nucleus, where it orchestrates the expression of target genes while regulating the transcription of other genes critical for cellular homeostasis and proliferation[10].High expression of Notch 3 ICD was detected in PASMCs isolated from patients with PH,these levels were proportional to PH severity, and inhibition of Notch 3 signaling inhibited the formation of hypoxia-induced PH[11]. Furthermore, inhibition of Notch 3 receptor cleavage using a monoclonal antibody targeting Jag1 ligand reversed hypoxia-induced PH in a rodent model[12].Onestudy has also reported that Notch 2 signaling promotes PASMC proliferation and consequent PH, while Notch 2 inhibition alleviates PH[13]. However, Jag2, which activates Notch 2 signaling by cleaving Notch 2 transmembrane receptors, has yet to be investigated in the pathophysiology of PH.

In this study, we aimed to investigate whether Jag2 is involved in the pathogenesis of PH and explore the underlying mechanisms. We report our findings that PASMCs-derived Jag2 contributes to PH formation by targeting the mitochondrial function and Sirt-1/Nrf2/HO-1 pathway. Moreover, sirtuine 1 (Sirt1) deficiency abolished protective effect of Jag2 inhibition.

## Methods

### Ethical approval

Six to eight-week-old, 180–240 g male Sprague-Dawley rats were purchased from the Wuhan Moubaili Biotechnology CO., LTD. All animals were kept at a constant temperature of 25°C in the Animal Laboratory Center, Renmin Hospital of Wuhan University, following a 12-hour light/dark cycle. The Renmin Hospital of Wuhan University Institutional Animal Care and Use Committee approved all animal experiments (IACUC Issue No. 20220723A).

### Animal model of chronic hypoxia and PH

To induce rats PH, the animals were subjected to a 4-week exposure of chronic hypoxia (10% O_2_) within a ventilated chamber. Control rats were maintained under normoxic conditions. Throughout the experimental period, all animals (6 rats per group) had unrestricted access to standard rat chow and water. At the end of the treatment, right ventricular systolic pressure (RVSP) and pulmonary vascular remodeling parameters were assessed as described below and tissue samples collected. After completing the measurement of RVSP, all rats were euthanized by inhalation of a sufficient amount of CO_2_ before proceeding with the collection of heart and lung tissue samples. To induce chronic persistent hypoxia in combination with gene transfer, rats were anesthetized with intraperitoneal injection of pentobarbital sodium (50 mg/kg). Subsequently, adeno-associated virus serotype 1-Jag2 (AAV1.jag2, sequence: 5’- GTCAGGCTCGGCGTTGATAC-3’; Vectorbuilder, China) or AAV1.Luc (sequence: 5’-TTCTCCGAACGTGTCACGTAA-3’) was administered via intratracheal route two weeks beforechronic hypoxia treatment. Analgesia was achieved through subcutaneous injection of 0.02 mg/kg of buprenorphine.

### Evaluation of right ventricular function

RVSP was directly detected via catheterization. A longitudinal skin incision is made on the right side of the neck to expose the right external jugular vein. Distal ligation is performed, and a loose knot is tied proximally to secure the catheter. A polyethylene catheter (filled with heparinized saline) is gradually inserted into the right ventricle through the incision made in the right external jugular vein. During the insertion process, continual adjustments are made, and the catheter is considered to have entered the right ventricle when waveform pressure curves are observed.Data acquisition and analysis were performed using the PowerLab data acquisition system (ADInstruments, Sydney, Australia) and LabChart 7.2 software. The ratio of the right ventricle to the combined weight of the left ventricle and septum (Fulton Index; [14]) was used to determine right ventricular hypertrophy.

### Histopathological examination of lung tissue and immunostaining

The left lungs were fixed in a 4% paraformaldehyde solution for 24 h, and the right lungs were removed and quickly frozen in liquid nitrogen for subsequenthomogenization. Distal pulmonary vascular were visualized in paraffin-embedded lung tissue sections (5 μm) using hematoxylin and eosin (H&E) staining and α-smooth muscle actin (α-SMA) staining to assess smooth muscle presence. Medial wall thickness of approximately 20 muscular arteries ranging from 20–50 μm and 50–100 μm in diameter was calculated using Image J as follows: medial wall thickness=(total vascular area ‐ lumen area)/total vascular area[15]. Masson’s trichrome staining was used to assess fibrosis and collagen deposition. Frozen sections and western blot were employed to detect the inhibitory effect of AAV1-Jag2. Immunohistochemical analysis with an anti-α-SMA antibody (1:400, Cell Signaling, 19245S) and immunofluorescence staining with an anti-Jag2 antibody (1:100, Santa Cruz, sc-515725) were conducted to detect α-SMA and Jag2 expression, respectively, in lung sections.

### Cell experiments

Rat PASMCs were acquired from the BeNa Culture Collection (Henan, China) and cultured to passagethree to five for following experiments. PASMCs were cultivated in Dulbecco’s modified Eagle medium-high glucose (Hyclone, Logan, UT) supplemented with 10% fetal bovine serum (Gibco, Thermo Fisher Scientific, Waltham. MA; 302220F). Transfection of Rat si-Jag2, si-Sirt1, or NC siRNA (si-Jag2: 5’- TGTCGCACCCTCTGGTATATG-3’; si-Sirt1: 5’- CTAGACCAAAGAATGGTATTT-3’; NC si-RNA: 5’-UUCUCCGAACGUGUCACGUTT-3’; Hanbio) into PASMCs was carried out using Lipofectamine 3000 (Invitrogen, Waltham, MA; L3000001) following standard procedures. After transfection for 24 h, the medium was replaced and transfection efficiency was verified by qPCR or western blotting. After 48 hours transfection, cells were used for anoxic experiments. The normal group cellswere incubated under standard conditions at 37°C with 5% CO_2_ and 21% O_2_, while the PASMCs exposed to hypoxia were cultured in culture dishes placed inside a hypoxia incubator with 5% CO_2_ and 1% O_2_. Following established protocols, the hypoxia duration for the in vitro model of hypoxia-induced PH was set at 24 hours.

### Identification andacquisition of RNA-seq data

RNA-seq data retrieved from the GEO database (GSE85618) were analyzed using the Limma package. Differentially expressed genes (DEGs) meeting the criteria of |log2-fold change| > 2 and P value < 0.05 were selected for further analysis.

### Cell proliferation assay

2×10^4^normal or si-Jag2 transfected PASMCs were seeded into 96-well plates and cultured overnight. Subsequently, the cells were subjected to either normoxia or hypoxia for a duration of 24 hours. Cell viability was evaluated at 450nm absorbance using the CCK-8 kit (Beyotime, Shanghai, China) following the provided instructions. The relative cell viability was determined by calculating the OD values at 450 nm from one separate experiments in the nomoxia control group.While proliferation was determined utilizing the EdU Cell Proliferation Assay Kit (Beyotime). Green staining indicated positive cells, and DAPI was applied as a blue nuclear counterstain. Fluorescence microscopy was employed to capture images, which were subsequently analyzed using ImageJ software.

### Flow cytometry evaluation of apoptosis

Treated PASMCs were cultured in six-well plates containing 10% fetal bovine serum. After 24 h, cells were harvested and subjected to staining with Annexin V-FITC and propidium iodide (PI) using a flow cytometry detection system as per the manufacturer’s instructions (BD Biosciences, Franklin Lakes, NJ).

### Analysis of mitochondrial membrane potential, mitochondrial reactive oxygen species, and detection of oxidative stress

Jag2- or Sirt1-inhibited PASMCs were incubated in six-well plates and treated under normoxic or hypoxic conditions. The mitochondrial membrane potential was assessed with the JC-1 kit (Solarbio Life Sciences, Beijing, China) according to standard procedure. Fluorescent images were acquired using a fluorescence microscope (Olympus, Tokyo, Japan), and mitochondrial membrane potential depolarization was quantified by calculating the percentage of red/green fluorescence using ImageJ software. The mitochondria-targetingsuperoxide anionindicatorMitoSOX (5 mM, Yeasen Biotechnology, Shanghai, China)was used to assay mitochondrial ROS(mtROS) formation.After staining for 15 min, cells were washed with PBS and acell suspensionproduced with trypsin.Finally,mtROS production was detected by flow cytometry and quantified using FlowJo software.To detect oxidative stress in lung tissue, *DHE* staining was performed according to the manufacturer’s instructions. SOD, MPO, and MDA levels were assessed with a SOD assay kit (Sigma-Aldrich, St. Louis, MO), MPO assay kit (Invitrogen), and MDA assay kit (Sigma-Aldrich) according to the manufacturers’ instructions.

### Western blotting

Protein extraction from lung tissue and PASMC lysates was performed using RIPA lysis supplemented with a protease inhibitor (PMSF) and cocktail (Servicebio, Wuhan, China).Protein concentrationswere quantified using a BCA kit (Beyotime), and 20–40μg of protein lysate were subjected tosodium dodecyl-sulfate polyacrylamide gel electrophoresis and transfer to poly(vinylidene fluoride) membranes (0.45 μm, MilliporeSigma, Burlington, MA). The membrane was then blocked with 5% skimmed milk and incubated overnight at 4°C with the respective primary antibodies: anti-Jag2 (1:1000, Santa Cruz Biotechnology, sc-515725), anti-Nrf2 (1:1000, Cell Signaling Technology, Danvers, MA, #12721), anti-HO1 (1:2000, Cell Signaling Technology, #26416), anti-Bax (1:1000, Cell Signaling Technology, #41162), anti-Bcl2 (1:1000, Proteintech, Rosemont, IL,68103-1-Ig), anti-Coxiv(1:5000, Proteintech,11242-1-AP), anti-Tom20 (1:3000, Proteintech, 11802-1-AP), anti-Sirt1 (1:2000, Proteintech, 13161-1-AP), and anti-GAPDH (1:10000, Abcam, ab8245), anti-PCNA (1:1000, ABclonal). Immunoblotting was carried out using the HRP-labelled secondary antibodies and detected with ECL System (Bio-Rad, Hercules, CA). Protein expression was further quantified via ImageJ and the ratio to GAPDH calculated.

### Quantitative real-time PCR

RNA extractionfrom lung tissues or PASMCs was performed using TRIzol(Takara Bio, Shiga, Japan). The quantity and quality of extracted RNA were determine using a Nanodrop ND-1000 spectrophotometer (ThermoFisher Scientific). For cDNA synthesis, 1 μg of RNA was used as a template with a cDNA cloning kit (BioFact™, BR123‐R10k). qPCR was conducted on a Bio-Rad CFX Connect Real-Time PCR Detection System (Bio-Rad).The internal reference of mRNA was *Actb*.The 2^(-ΔΔCt) methods were used to determine the relative expression levels of the target genes.Table 1 lists the PCR primer sequences.

**Table 1:** Primers for real-time PCR (rat).

Gene name	Forward	Reverse
*Jag2*	CGCCAACTGCCACATCAATATCAAC	AAGCCATCCACCAGGTCCTCAC
*Sirt1*	ACGCCTTATCCTCTAGTTCCTGTGG	CGGTCTGTCAGCATCATCTTCCAAG
*Sirt2*	CCACGGCACCTTCTACACATCAC	ATCGGGCTTTACCACATTCTGACAC
*Sirt3*	TCAGCAGTATGACATCCCGTACCC	GTGAAGCAGCCGAAGGAAGTAGTG
*Sirt4*	GGACTCTACGCCCGCACTGAC	GGCTGGTGAGAGGAGAACTGAGG
*Sirt5*	CGGTGTACCTCGTGTGGCAATG	GTTTGTGGACTGGGATTCTGGACTC
*Sirt6*	GGCTACGTGGATGAGGTGATGTG	GCTGTCGGGCTTGGGCTTATAG
*Actb*	ACCCCATTGAACACGGCATT	CTCATAGAAGAGAGTCCTGGGTCA

### Statistical analysis

GraphPad Prism software (CA, USA) was used to evaluate the significance of differences between two groups using Student’s t-test and among multiple groups using one-way ANOVA followed by Tukey multiple comparison test for parametric variables and the Kruskal Wallis test for nonparametric variables. Data in the present study are presented as the mean ± standard deviation values and were obtained from triplicate experiments. P<0.05 was considered to indicate a significant difference.

## Results

### Jag2 is upregulated in hypoxia-treated rats and PASMCs

**Fig 1:**
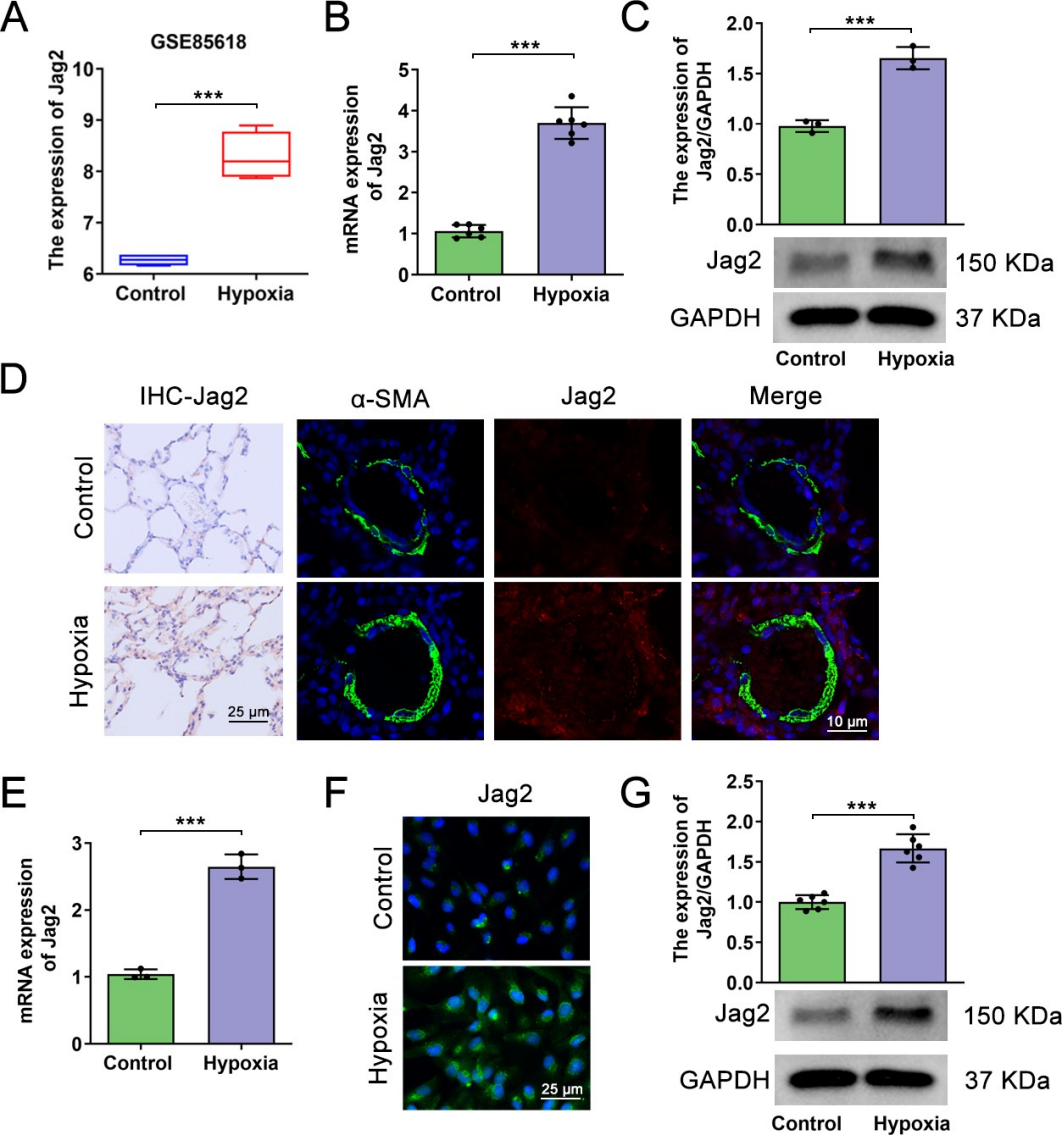
Jag2 expression is upregulated in hypoxia-treated rats and PASMCs. (A) Visualization of *Jag2* expression in the GSE85618 dataset. (B) Jag2 mRNA and protein (C) expression by qRT-PCR, immunohistochemistry, and western blotting in lung tissue of normal and hypoxia-treated rats. N = 6 rats per group. (D) Immunofluorescence double staining of α-SMA and Jag2 in lung tissues of normal and hypoxia-treated rats. Scale bar, 10 μm. (E) *Jag2* mRNA and protein expression by qRT-PCR, immunofluorescence (F), and western blotting (G) in normal and hypoxia-treated PASMCs. Each group N = 3, and three biological replicates per group for cellular experiments. Scale bar, 25 μm. Data shown are mean ± SD; ***P < 0.001.

The publicly-available GSE85618 dataset was first interrogated to determinegenes differentiallyexpressed under hypoxic conditions. Following normalization, it was determined that *Jag2*was differentially expressed in hypoxic rats or PASMCs (**S1A and S1B Fig, for Jag2, LogFC = 2.018, P < 0.001**) and may therefore play a role in chronic hypoxia-associated vascular adaptations.*Jag2* was significantly upregulatedby sustained hypoxia compared with normoxia in the GSE85618 dataset (**Fig 1A**). Furthermore, in our experimental rat models of hypoxia-induced PH, both mRNA and protein levels of Jag2 were significantly upregulated in lung tissue(**Fig 1B–1D**).Immunofluorescence double staining of α-SMA and Jag2 showed that α-SMA expression was more pronounced in hypoxia-treated pulmonary arteries, while Jag2 was increased in pulmonary arteries as well as in surrounding tissues.These results were further corroborated by similar findings in hypoxia-induced ratPASMCs*in vitro* (**Fig 1E–1G**).

### Jag2 inhibition effectively ameliorates hypoxia-induced rats PH

**Fig 2:**
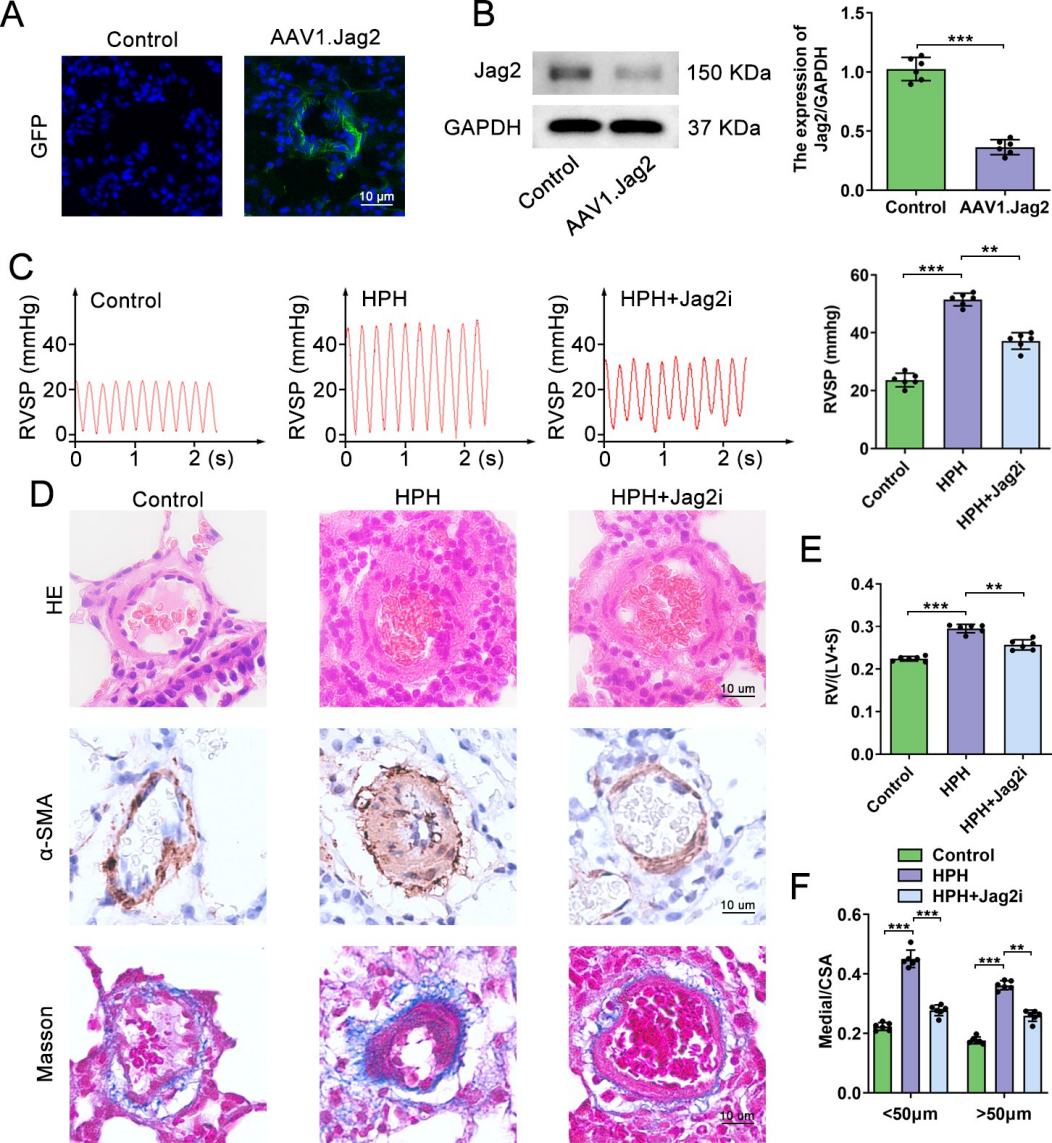
Inhibition of Jag2 effectively ameliorates hypoxia-induced PH in rats. (A) Fluorescence microscopy images of lung tissue four weeks after intratracheal AAV.1Jag2 instillation, where green fluorescence represents the expression and localization of AAV1 in lung tissue. DAPI stains the nuclei blue. Scale bar=10μm. (B) Representative immunoblot images and quantitative analysis of Jag2 protein expression in the control and AAV1.Jag2 groups. (C) Waveform diagram and quantitative analysis of RVSP (mmHg) in the indicated groups. (D) Representativemicroscopic images of distal pulmonary vascular stained with H&E, immunostained for α-SMA, and Masson trichrome in control, HPH, and HPH+Jag2i rats. Scale bar=10μm. (E) Fulton index RV/(LV+S) in the three groups. (F) The relative medial thickness expressed as a ratio of (total vascular area ‐ lumen area) to total vascular area (media/CSA). N=6 rats per group. Data shown are mean ± SD; **P < 0.01, ***P < 0.001.

In order to assess Jag2 elimination efficacy in a hypoxia-induced PH rat model, rats were divided into controls (Control, AAV1 encoding luciferase), HPH (hypoxia-induced PH), or HPH+Jag2i (hypoxia + AAV1 encoding rat Jag2). Two weeks after intratracheal instillation of either AAV1.Jag2 or AAV1-luciferasecontrols, rats were subjected to either normoxia or sustained hypoxia for four weeks. *Jag2* knockdown was validated by quantifying fluorescence in frozen lung tissue sections and western blotanalysis the expression of Jag2 four weeks after AAV1 instillation. Compared with controls, the AAV1.Jag2-transfected group expressed eGFP protein in the lungs and Jag2 protein levels was significantly suppressed (**Fig 2A and 2B**), confirming the efficacy of intratracheal instillation with AAV1.Jag2.Four weeks following continuous hypoxia exposure, RVSP, peripheral pulmonary vascular remodeling, perivascular pulmonary fibrosis, and RV hypertrophy were all significantly increased compared with normoxic controls.Treatment with AAV1.Jag2 significantly improved mean RVSP (**Fig 2C** 36.8±3.033 vs. 51.8±4.245 mmHg, P<0.001) and distal pulmonary vascular remodeling (**Fig 2D–2F** Medial thickness/CSA: 0.276±0.020 vs. 0.451±0.033, P<0.001, <50μm; 0.279±0.053 vs. 0.363±0.017, P = 0.009, >50μm) compared with the HPH group. Furthermore, Jag2 attenuation diminished RV hypertrophy, as measured by the Fulton index (**Fig 2E** 0.258±0.013 vs. 0.294±0.011, *P* = 0.0014). Masson’s trichrome staining revealed a reduction in perivascular pulmonary fibrosis in the HPH+Jag2i group (**Fig 2D**). Furthermore,it has beendiscerned that suppression of Jag2 expression results in a concomitant decrease in the heightened expression of vascular remodeling indicators, including α-SMA and PCNA (**S2A Fig**), in response to hypoxia. These results highlight the potential therapeutic value of Jag2 inhibition for hypoxia-induced pulmonary hypertension.

### Absence of Jag2 alleviates hypoxia-induced oxidative stress injury in rats

**Fig 3:**
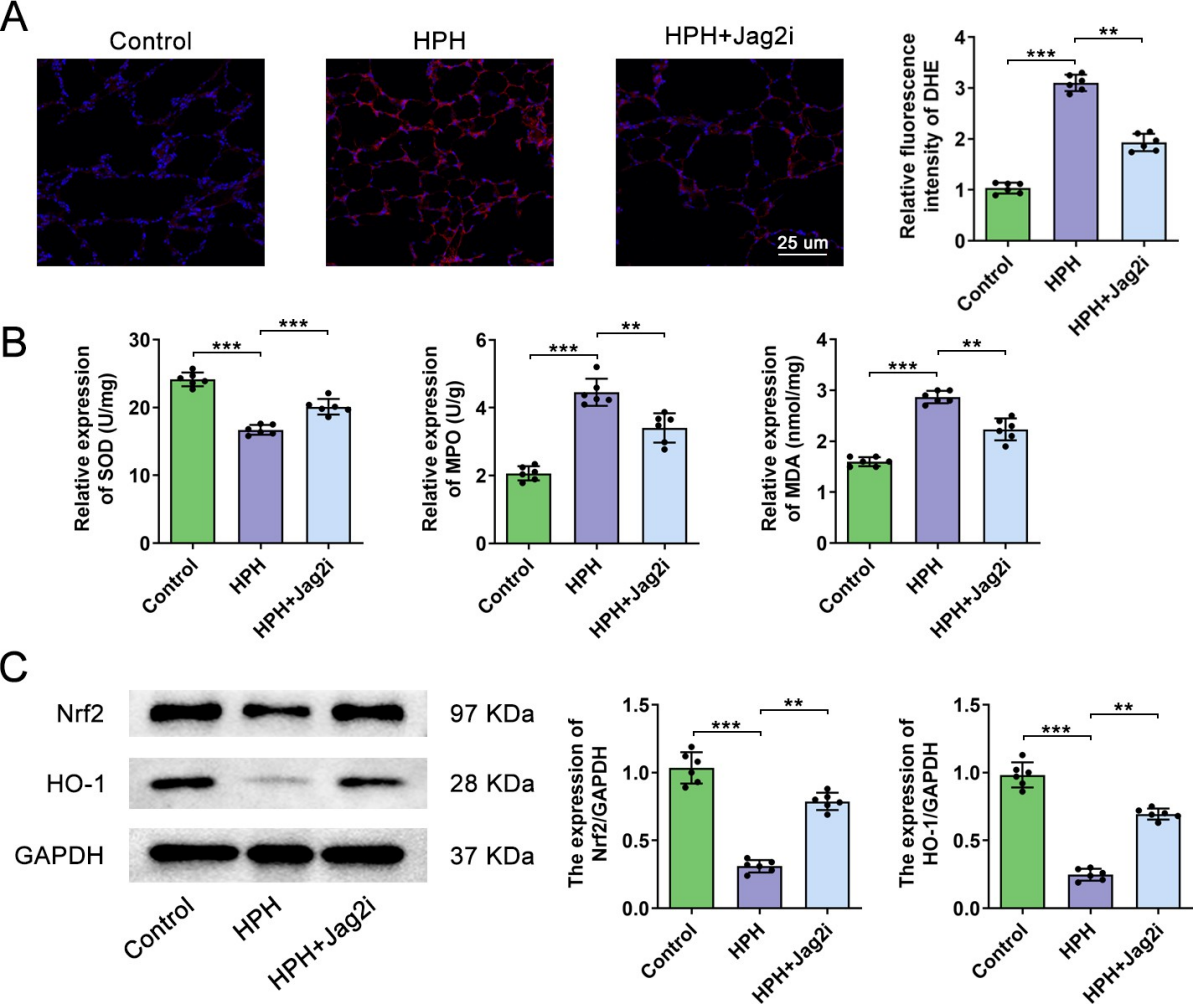
Absence of Jag2 alleviates hypoxia-induced oxidative stress injury in rats. (A)Comparison of DHE staining and quantitative analysis in rat lung tissue samples treated with Control, HPH, and HPH+Jag2i. (B) Comparison of lung SOD, MPO, and MDA activity in the different groups. (C) Representative immunoblot images and quantitative analysis of Nrf2 and HO-1 protein expression inControl, HPH, and HPH+Jag2i rats. N=6 rats per group. The values expressed are mean ± SD. *P< 0.05, **P <0.01, ***P < 0.001.

DHE, SOD, MPO, and MDA are markers of oxidative stress and inflammation[16]. Compared with controls, lung DHE, MPO, and MDA activities were significantly increased in the HPH group, while SOD activity significantly decreased (**Fig 3A and 3B**). However, administration of AAV1.Jag2 decreased lung DHE, MPO(3.388±0.482 vs. 4.504±0.428, *P* = 0.047), and MDA (2.26±0.230 vs. 2.86±0.134, *P* = 0.001)activities and increased SOD (20.20±1.263 vs. 16.82±0.756, *P*<0.001)activity (**Fig 3A and 3B**).Nrf2 and HO-1 are key components of the cellular defense system, and their activation is an important therapeutic approach in oxidative stress-related diseases[17]. In the control group, Nrf2 and HO-1protein expression were maintained at high levels (**Fig 3C**). Hypoxia significantly reducedNrf2 and HO1 levels, suggesting an imbalance in oxidation and anti-oxidation. More importantly, Nrf2 and HO-1levels significantly increased when AAV1.Jag2 was administered (**Fig 3C**).

### Jag2 deficiency prevents proliferation and promotes apoptosis in hypoxia-treated PASMCs

**Fig 4:**
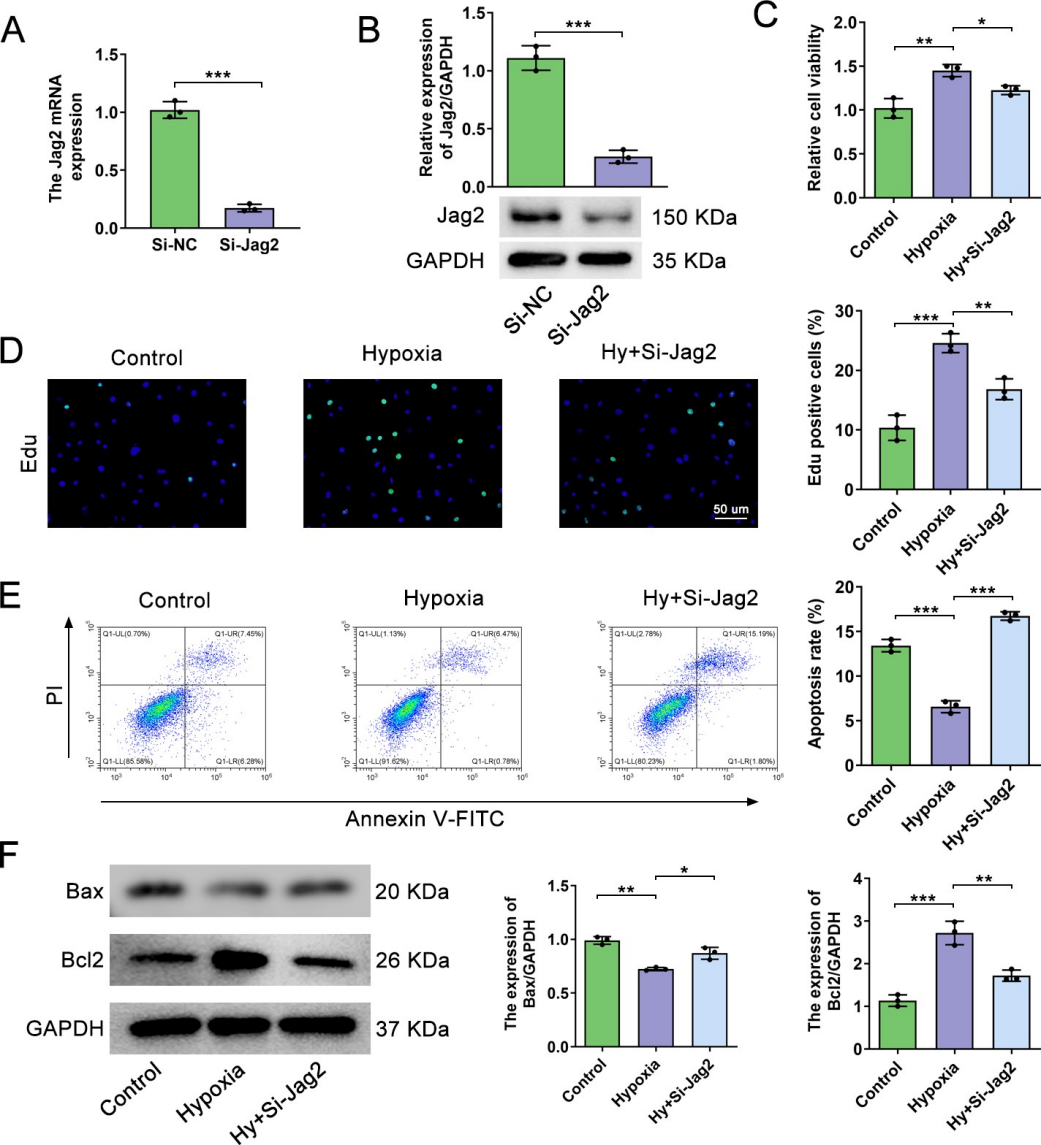
Jag2 deficiency prevents proliferation and promotes apoptosis in hypoxia-treated PASMCs. (A) PASMCs were transfected with Si-NC or Si-Jag2 and analyzed by qRT-PCR and western blotting (B). (C) PASMC viability in control, hypoxia, and hypoxia+Si-Jag2 groups was detected by the CCK8 assay. (D) EdU analysis of PASMCs in the indicated groups. (E) Flow cytometry and quantitative analysis of apoptosis in the three groups. (F) Representative western blots and quantitative analysis of Bax/Bcl2 in the three groups. *P<0.05, **P<0.01, ***P<0.001, each group N = 3. Three biological replicates per group for cellular experiments.

To explore the potential biological role of Jag2 in hypoxia-treated PASMCs, PASMCs were transfected with Si-*Jag2*as validated byqRT‒PCR and western blotting (**Fig 4A and 4B**). With respect to cell viability (CCK8 assay), inhibition of Jag2 in hypoxia-treatedPASMCs significantly decreased cell viability (**Fig 4C** 1.227±0.051 vs. 1.45±0.07, *P* = 0.012). These results were confirmed with theEdUassay, which revealed a reduction in the number of EdU-positive cells in the Jag2-inhibited group compared withhypoxic controls (**Fig 4D**). Moreover, Jag2 deficiency significantly increased apoptosis compared with the hypoxia group by flow cytometry (**Fig 4E** 16.733%±0.724% vs. 6.56%±0.668%, *P*<0.001). Jag2 deficiency also increased expression of the pro-apoptotic protein Bax and inhibited expression of the anti-apoptotic protein Bcl2 (**Fig 4F**).Together, these results show that Jag2may play a crucial role in regulating PASMC function under hypoxic conditions.

### Jag2 knockdown ameliorates hypoxia-induced mitochondrial dysfunction

**Fig 5:**
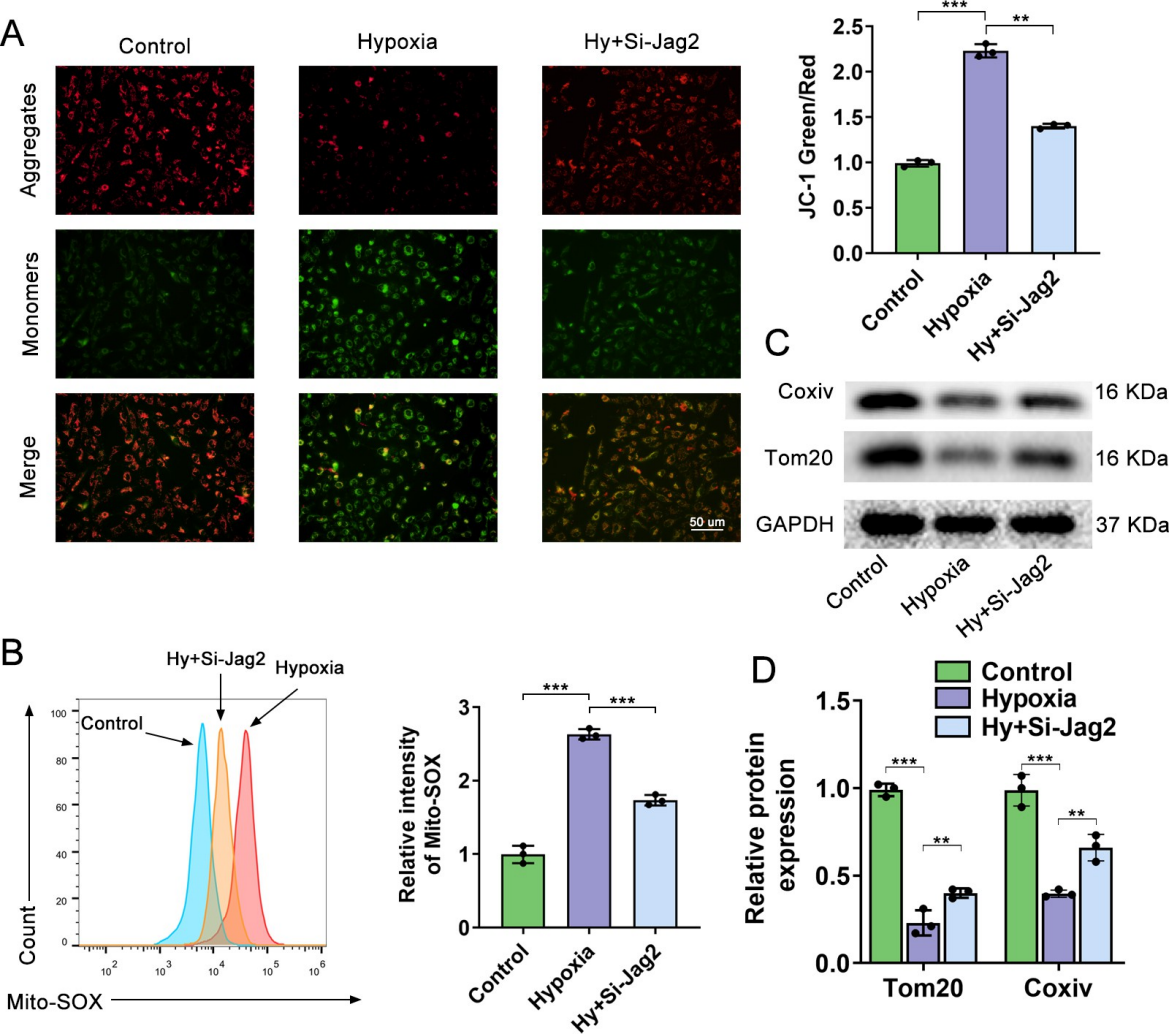
Jag2 knockdown ameliorates mitochondrial dysfunction induced by hypoxia. (A) JC-1 staining of the intracellular mitochondrial membrane potential of control, hypoxia, and hypoxia+Si-Jag2 groups. JC-1 aggregates produce red fluorescence; JC-1 monomer produces green fluorescence. An increase in the relative ratio of red to green fluorescence indicates mitochondrial depolarization. (B) Intracellular mitochondrial superoxide levels in the three groups were tested using the MitoSOX assay and flow cytometry. (C) Representative western blots and quantitative analysis (D) of Coxiv and Tom20 in three groups. *P<0.05, **P<0.01, ***P<0.001, each group N = 3. Three biological replicates per group for cellular experiments.

Hypoxia is known to induce mitochondrial dysfunction[18]. Consistent with this,hypoxia-induced mitochondrial dysfunction was also observed. As shown in **Fig 5A**, compared with control cells, hypoxia increasedthe green to red fluorescence ratioafter JC-1 staining,indicative of mitochondrial membrane potential depolarization, while Jag2 inhibitionreversed this trend.MitoSOX is a fluorescent dye commonly used to measure mitochondrial superoxide. As shown in **Fig 5B**, Jag2 inhibition decreased MitoSOXlevels compared with the hypoxia group. Moreover, Jag2 deficiency prevented the degradation of mitochondrial proteins Coxiv and Tom20 induced by hypoxia (**Fig 5C and 5D**). Overall, Jag2 deficiency ameliorated the mitochondrial dysfunction induced by hypoxia.

### Jag2 regulates mitochondrial injury in hypoxia-induced PASMCs via Sirt1

**Fig 6:**
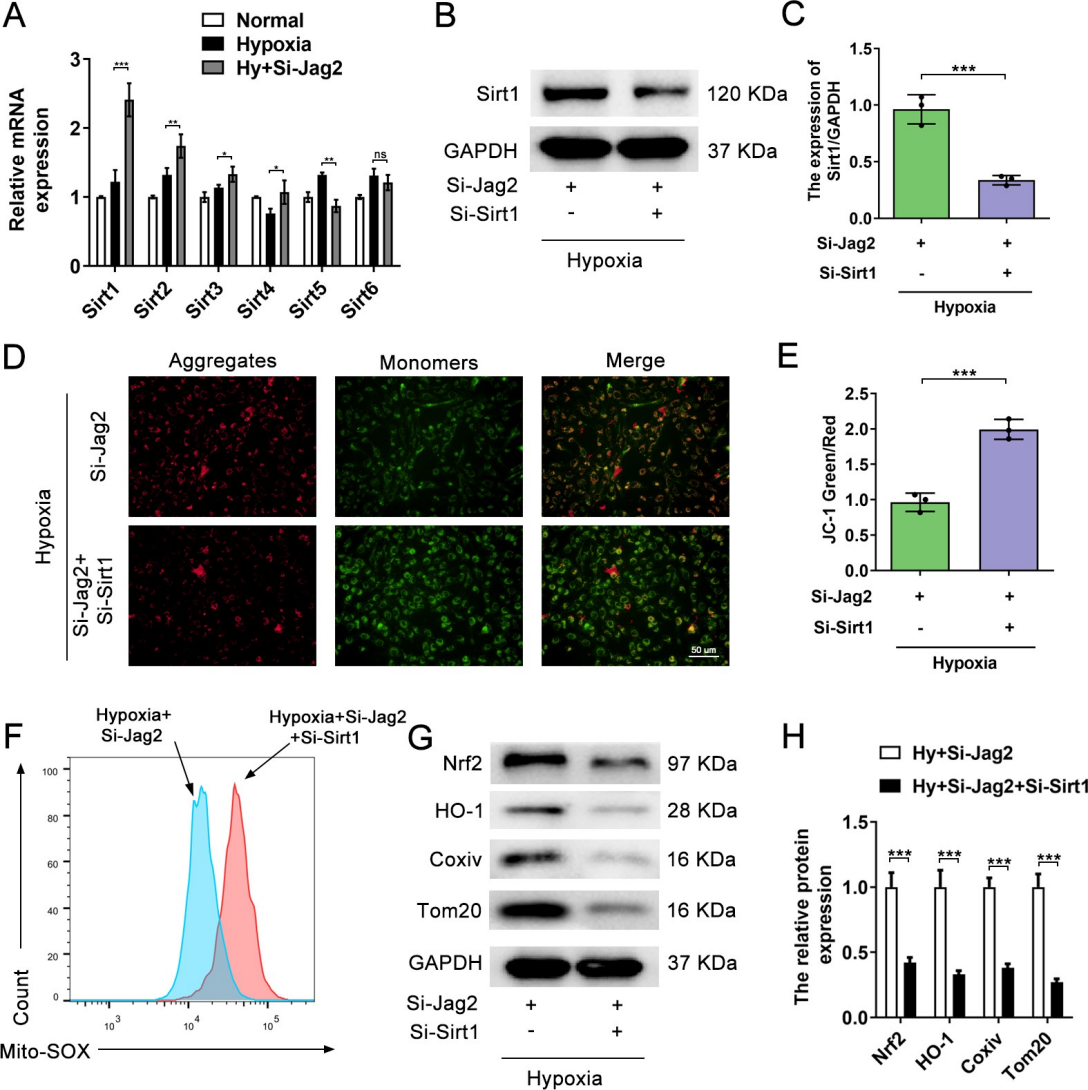
Jag2 regulates mitochondrial injury in hypoxia-induced PASMCs via Sirt1 signaling. (A)Comparison of Sirt1-6 mRNA expression between control, hypoxia, and hypoxia+Si-Jag2 groups by qRT‒PCR. (B) Representative western blots and quantitative analysis (C) of Sirt1 protein expression in hypoxia+Si-Jag2 and hypoxia+Si-Jag2+Si-Sirt1 groups. (D) JC-1 staining and quantitative analysis (E) of the intracellular mitochondrial membrane potential in the indicated groups. (F) Intracellular mitochondrial superoxide levels of the two groups were tested using the MitoSOX assays and flow cytometry. (G) Representative western blots and quantitative analysis (H) of Coxiv and Tom20 in the two groups. *P<0.05, **P<0.01, ***P<0.001, each group N = 3. Three biological replicates per group for cellular experiments.

Given that Sirt signaling has been shown to regulate mitochondrial injury in hypoxia and that the Notch and Sirt signaling pathways interact[19, 20], we hypothesized that Jag2 may regulate mitochondrial injury in hypoxia-induced PASMCs via Sirt signaling. To investigate this hypothesis, qRT-PCR was performed to detect mRNA expression of *Sirt1-6* in cells under normoxic, hypoxic, and hypoxia+Si-*Jag2* conditions. Hypoxia slightly increased *Sirt1*, *Sirt2*, *Sirt3*, *Sirt5*, and *Sirt6* expression and, compared with the other Sirt proteins, Si-*Jag2* significantly increased expression of *Sirt1* (**Figs 6A and S2C**).Sirt1 transfection efficiency was confirmed (**Figs 6B and 6C and S2B**).The results of the JC-1 assay showed that a decrease in *Sirt1*in PASMCs significantly increased the green to red fluorescence ratioin *Jag2*-downregulated cells (**Fig 6D and 6E**). The MitoSOXassay also revealed the same results, with an increase in MitoSOX levels observed after *Sirt1*inhibition in *Jag2*-downregulated cells (**Fig 6F**). *Sirt1*inhibition also promoted degradation of the mitochondrial proteins Coxiv and Tom20 after *Jag2*deficiency under hypoxic conditions(**Fig 6G–6H**). Together, these results illustrate that Sirt1inhibition abrogates the phenotypic alterations caused by Jag2 downregulation.

## Discussion

Here we demonstrated that Jag2 deficiency effectively attenuates pulmonary arterial pressure and pulmonary vascular resistance through activation of Sirt1 signaling and a subsequent reduction in hypoxia-induced PASMCmitochondrial dysfunction and proliferation. Our study for the first time presents compelling evidence that Jag2 is activated during the development of PH.Furthermore, our findings reveal a potentially promising new therapeutic strategy for PH, as we demonstrate that Jag2 inhibition can reverse the pathological antiapoptotic and proliferative phenotype of PASMCs.

The main protagonist in PH is pulmonary vascular remodeling, characterized by a thickening of the vascular intima and media, which is widely believed to arise through hypertrophy, proliferation, migration, and extracellular matrix deposition of various cell types including endothelial cells, smooth muscle cells, fibroblasts, inflammatory cells, and platelets[21].Previous studies have demonstrated a constant level of the Notch ICD in the small pulmonary arteries of patients with PH. Additionally, cleavage of the Notch receptor induces proliferation of PASMCs and confers anti-apoptotic properties [11]. The cis-activation of NOTCH signaling by JAG and DLL ligands has been demonstrated in fetal pulmonary artery development [22], several mammalian cell lines, including neural stem cells and fetal pulmonary artery development cells [23]. Additionally, it has been found that cleavage of the NOTCH receptor induces proliferation of PASMCs and confers anti-apoptotic properties. Meanwhile, both in the mouse model of hypoxia-induced PH and the rat model of MCT-induced PH,there is a notable and gradual increase in Notch1,Notch3,and Jagged1 expression within pulmonary artery walls,whichsuggests rapid activation of Notch signaling and expression during the development of PH[8].While previous studies have shown that the increase in expression of Notch pathway components, including Notch1, stimulatesthe proliferation of pulmonary artery smooth muscle cells [24], the mechanisms by which Notch ligand signals, particularly Jag2, regulate pathwaysof PASMC proliferation remain unknown.

Jag2, a transmembrane protein, is a critical player in the Notch signaling pathway. As a ligand for Notch receptors, Jag2 participates in numerous cellular processes including development, differentiation, and cell fate determination [25]. In addition to its involvement in normal physiological processes, Jag2is implicated in various diseases including cancer and cardiovascular disorders [26, 27]:Jag2 is upregulated in cancer cells and promotes tumor growth and progression by activating Notch signaling pathways [27], whilein cardiovascular disorders, Jag2 is involved in the regulation of vascular smooth muscle cell proliferation, migration, and angiogenesis [28]. Dysregulation of Jag2 expression has been observed in PH and vascular regeneration [29, 30], highlighting its potential as a therapeutic target.Our studies now reveal that Jag2 is highly expressed in lung tissue and PASMCsin hypoxia-induced PH models. In addition, inhibiting Jag2 expression alleviates hypoxia-induced oxidative stress damage and mitochondrial dysfunction.

Hypoxia triggers mitochondrial damage, leading to oxidative stress, inflammation, and pulmonary vascular remodeling. Mitochondrial dysfunction in PH is characterized by decreased mitochondrial biogenesis, altered mitochondrial dynamics, and the accumulation of mitochondrial ROS [31]. Moreover, therapeutic strategies that target mitochondrial dysfunction have shown promise in the treatment of PH. Sirt1 is a nicotinamide adenine dinucleotide-dependent protein deacetylase that plays an essential role in the regulation of mitochondrial function by deacetylating numerous mitochondrial proteins, including components of the electron transport chain and mitochondrial biogenesis regulators[32]. Dysregulation of Sirt1 is implicated in the pathogenesis of various diseases including PH[19].It was recently shown that Notch and Sirt1 signaling interact; indeed,notoginsenoside R1 significantly restored cerebral blood flow, improved mitochondrial energy metabolism, and promoted vascular regeneration by activating the NAMPT-NAD^+^-SIRT1 pathway and inhibiting Notch signaling after ischemia-reperfusion injury in rats, while Sirt1 inhibition partially reversed the modulatory effect of notoginsenoside R1 on Notch signaling [20]. Furthermore, Sirt1 activation successfully reversed the increase in liver sinusoidal endothelial cells senescence and restored the abnormal liver function induced by Notch activation, demonstrating that Notch drives liver sinusoidal endothelial cell senescence and liver homeostasis imbalance in a Sirt1-dependent manner [33]. For these reasons, we hypothesized that Jag2 regulates hypoxia-induced mitochondrial damage via Sirt1 in PASMCs.Consistent with this, we observed that Jag2 inhibition significantly increased the expression of Sirt1 but notSirt2-6, and the protective effect of Jag2 deficiency on hypoxia-induced mitochondrial damage was eliminated upon simultaneous inhibition of Sirt1.

Non-specific NOTCH inhibitors have been assessed in early-stage clinical trials for treating various malignancies, including chronic myeloid leukemia, chronic lymphocytic leukemia, T-cell lymphoblastic leukemia, as well as advanced solid tumors such as breast cancer and pancreatic cancer. However, the use of non-specific NOTCH inhibitors can lead to different side effects due to the inhibition of other NOTCH receptors, including refractory nausea, vomiting, abdominal distension, anorexia, and dehydration [34]. Hence, as the field of NOTCH inhibitor pharmacology advances, there is a growing anticipation for the development of a novel monoclonal antibody targeting specifically the JAG-2 ligand binding to NOTCH. This approach would effectively impede downstream NOTCH signaling, while circumventing the detrimental side effects associated with non-specific NOTCH receptor inhibition. Such a targeted therapeutic strategy holds great promise for addressing the complexities of PH, offering a potential breakthrough in its treatment paradigm.

This research has several limitations, not least the use of only rat PASMCs and not including pulmonary artery endothelial cells and lung fibroblasts, among others. Furthermore, due to the experimental constraints, this study did not utilize Jag gene-edited mice, and intratracheal injection of adenoviruses may also affect bronchial epithelial cells and alveolar epithelial cells.Furthermore, further investigation is needed to elucidate the specific functions of Jag2 in regulating mitochondria.Finally, the reversal and therapeutic effects of Jag2 inhibition on established PH were not explored.

## Conclusion

In conclusion, our study offers preclinical data supportinga new therapeutic approach for attenuating multiple pathological processes associated with PH.Moreover, Jag2 inhibition not only inhibitedPASMC proliferation but also restored hypoxia-induced oxidative stress injury and mitochondrial dysfunction. These findings highlight the enormous role of Jag2in promoting the development of PH and related disorders.

## Supporting information

S1 Fig Heat map and volcano plot of the GSE85618 dataset. (A) A visual representation in the form of a heat map to highlight the top 40 genes that show significant differences in expression levels within the GSE85618 dataset. Each column in the heat map corresponds to a particular sample, while each row represents the expression level of a specific gene. (B) volcano plot of the GSE85618 dataset showing the fold change (x-axis) differentially expressed genes.
(TIF)

S2 Fig Immunoblot images of α-SMA, PCNA and Sirt1. (A) Representative immunoblot images and quantitative analysis of α-SMA and PCNA protein expression in Control, HPH, and HPH+ Jag2i rats. N=6 rats per group. (B) Representative western blots and quantitative analysis of Sirt1 protein expression in Control and Si-Sirt1 groups. (C) Representative western blots and quantitative analysis of Sirt1 protein expression in Control, Hypoxia and Hy+Si-Jag2 groups. *P<0.05, **P<0.01, ***P<0.001. Three biological replicates per group for cellular experiments.
(TIF)

S1 Graphical abstract Hypoxia induces high intracellular Jag2 expression. And Jag2 activates intracellular intracellular structural domain, ICD transferred to the nucleus to regulate the expression of mitochondria-related gene Sirt1 through the Notch receptor recognition. It causes mitochondrial dysfunction and resistance to proliferation and apoptosis of PASMCs, which in turn promotes the occurrence and development of pulmonary hypertension.
(TIF)

S1 File Raw image of western blots. Original Images for blots.
(ZIP)

## Abbreviations

PH: Pulmonary hypertension
PASMCs: pulmonary artery smooth muscle cells
Jag2: Jagged 2
RVSP: right ventricular systolic pressure
ICD: intracellular structural domain
ROS: reactive oxygen species
mtROS: mitochondrial ROS
α-SMA: α-smooth muscle actin
H&E: hematoxylin and eosin
DEGs: Differentially expressed genes
PI: propidium iodide
Sirt1: Sirtuine 1
