# Supplementary figures and images for: Jagged 2 inhibition attenuates hypoxia-induced mitochondrial damage and pulmonary hypertension through Sirtuin 1 signaling
Source: PLoS One. 2024 Jan 26;19(1):e0297525. doi: 10.1371/journal.pone.0297525 (PMC10817012; doi:10.1371/journal.pone.0297525)

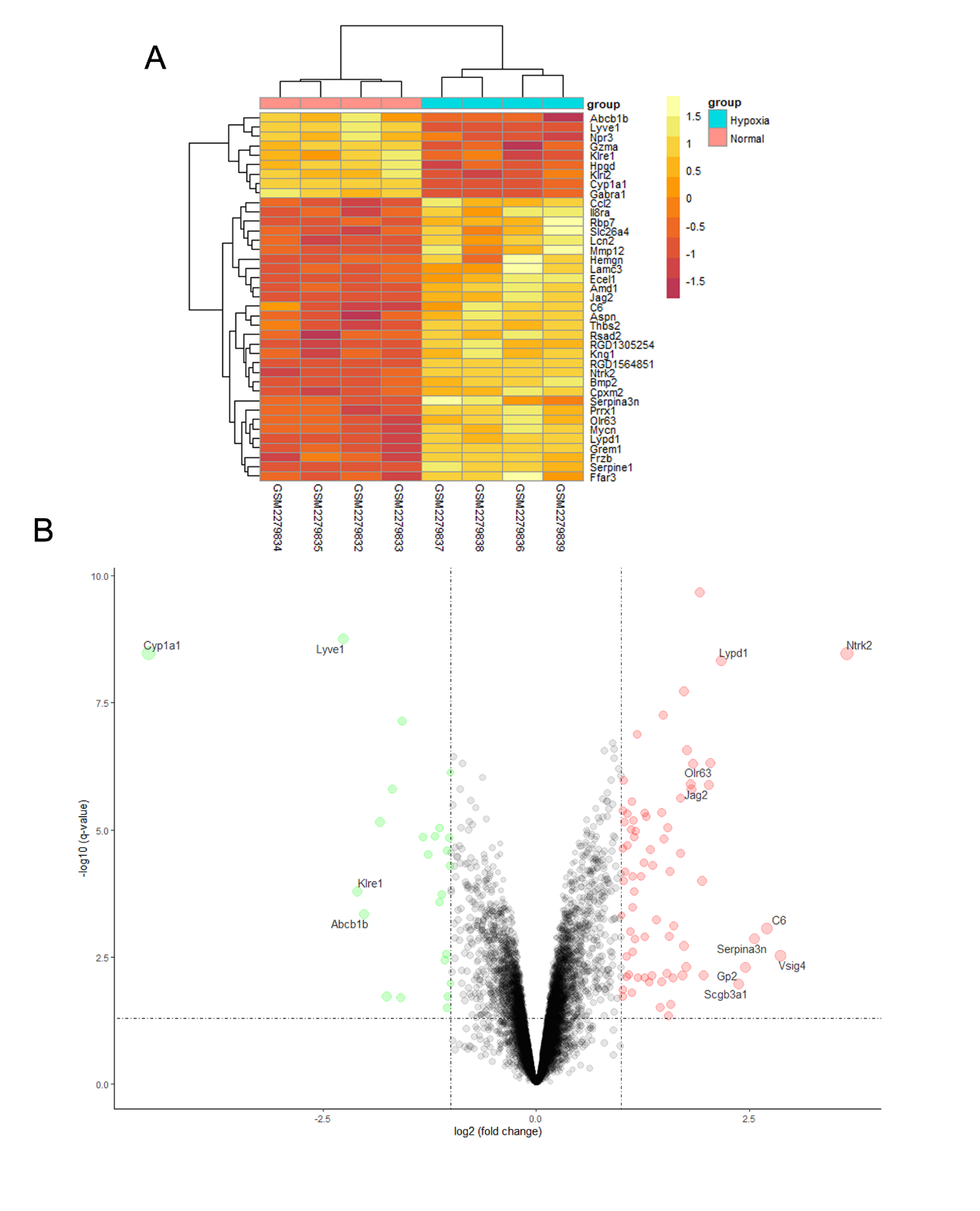

Supplement: S1 Fig — (A) A visual representation in the form of a heat map to highlight the top 40 genes that show significant differences in expression levels within the GSE85618 dataset. Each column in the heat map corresponds to a particular sample, while each row represents the expression level of a specific gene. (B) volcano plot of the GSE85618 dataset showing the fold change (x-axis) differentially expressed genes. (TIF) [file pone.0297525.s001.tif]

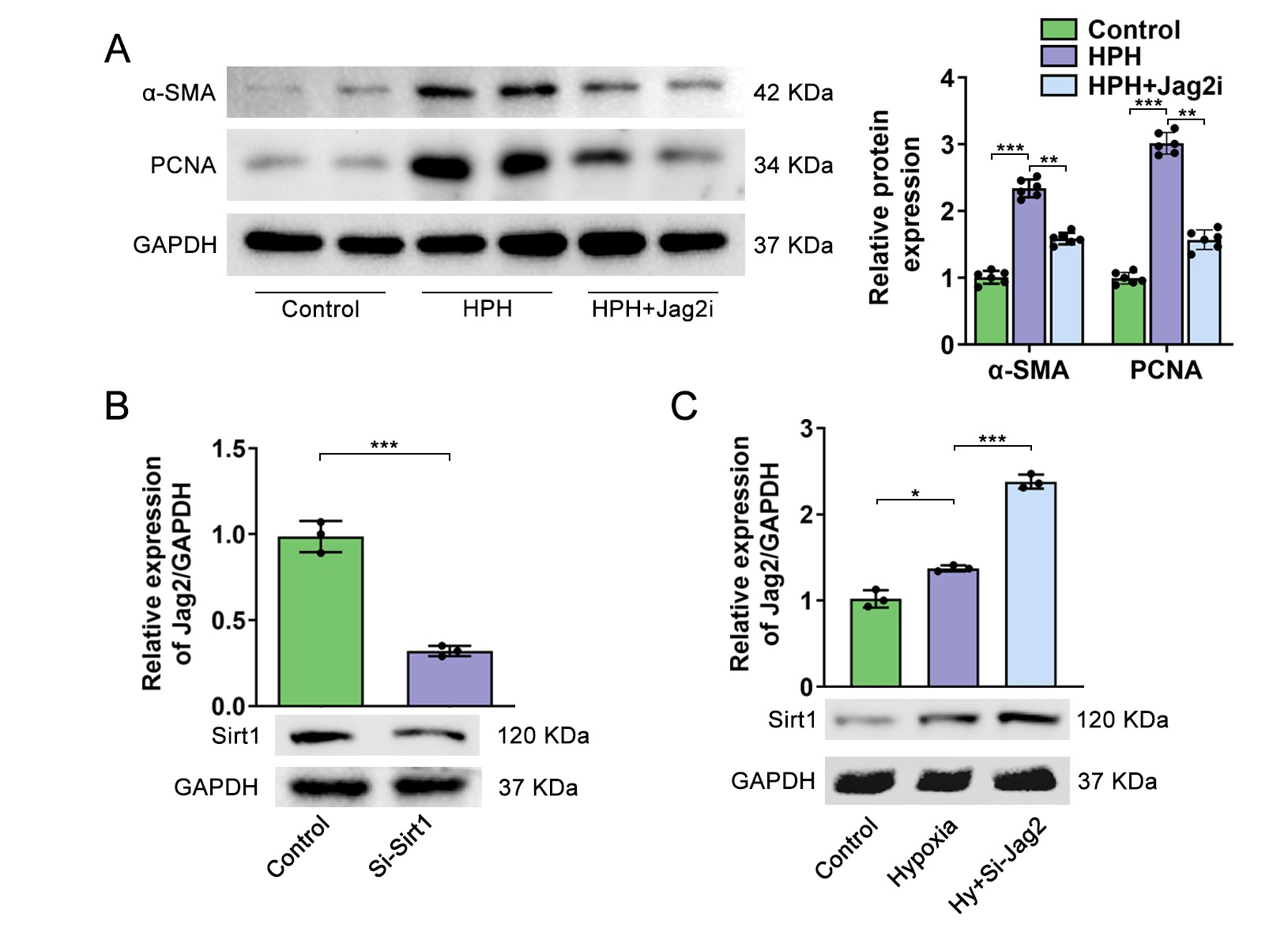

Supplement: S2 Fig — (A) Representative immunoblot images and quantitative analysis of α-SMA and PCNA protein expression in Control, HPH, and HPH+ Jag2i rats. N=6 rats per group. (B) Representative western blots and quantitative analysis of Sirt1 protein expression in Control and Si-Sirt1 groups. (C) Representative western blots and quantitative analysis of Sirt1 protein expression in Control, Hypoxia and Hy+Si-Jag2 groups. *P<0.05, **P<0.01, ***P<0.001. Three biological replicates per group for cellular experiments. (TIF) [file pone.0297525.s002.tif]

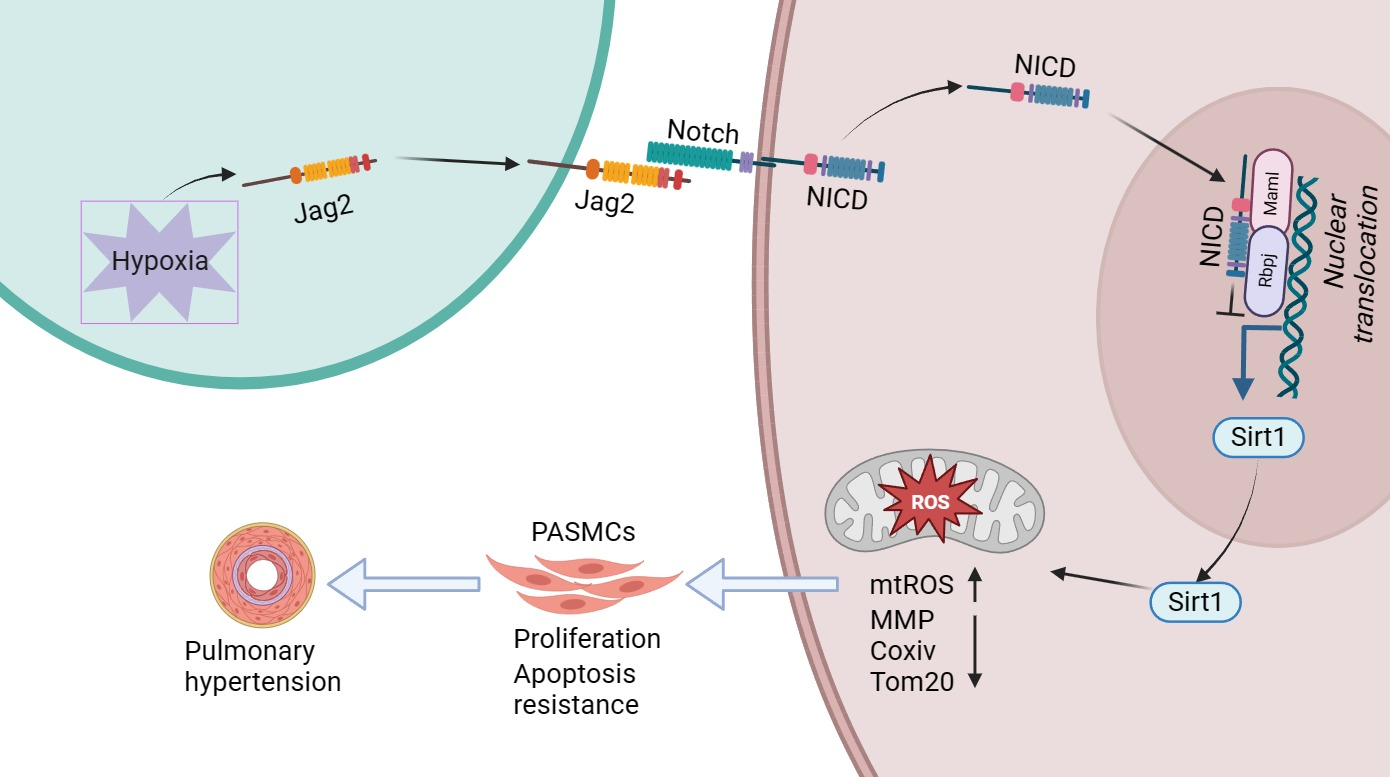

Supplement: S1 Graphical abstract — And Jag2 activates intracellular intracellular structural domain, ICD transferred to the nucleus to regulate the expression of mitochondria-related gene Sirt1 through the Notch receptor recognition. It causes mitochondrial dysfunction and resistance to proliferation and apoptosis of PASMCs, which in turn promotes the occurrence and development of pulmonary hypertension. (TIF) [file pone.0297525.s003.tif]

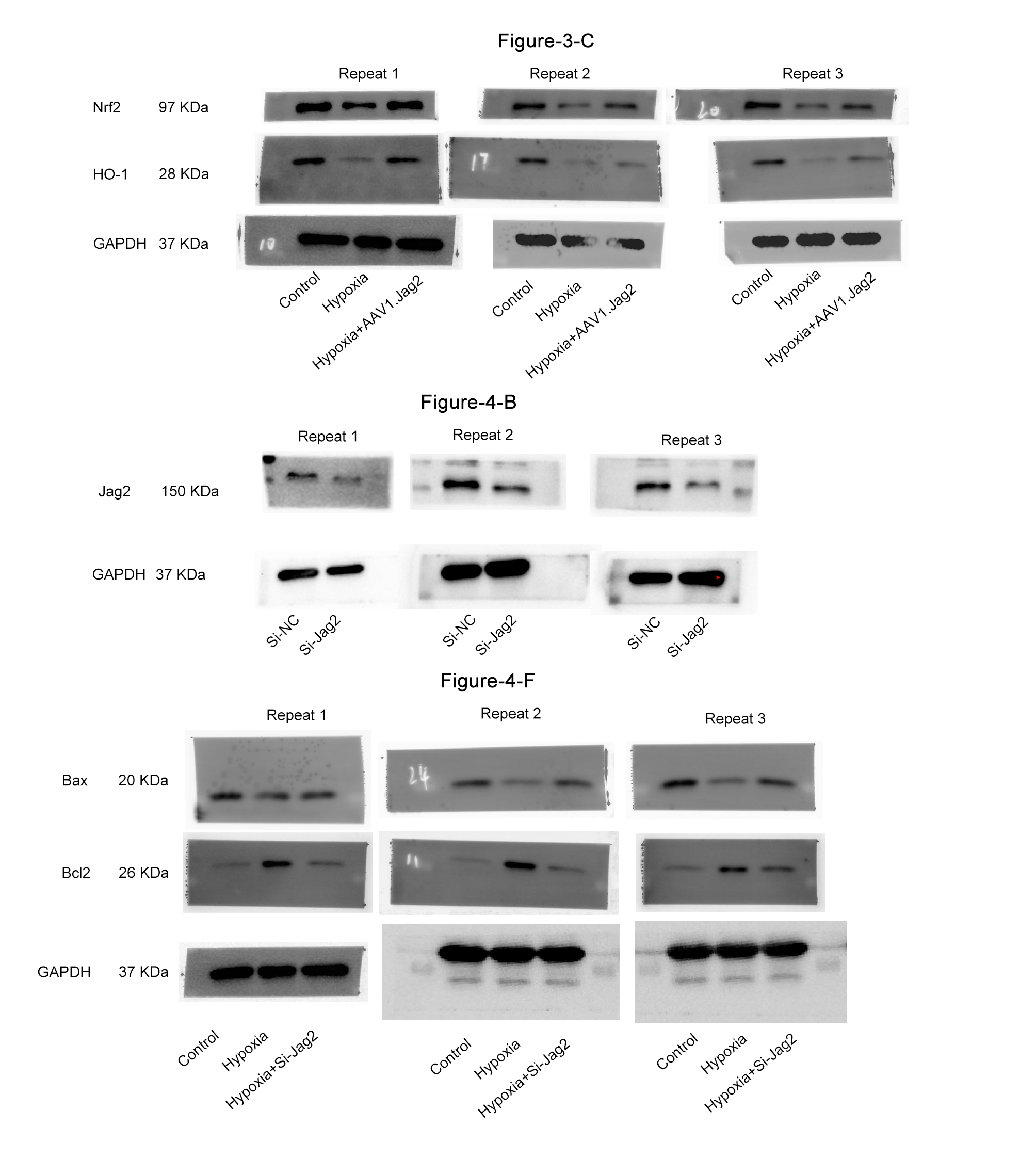

Supplement: S1 File — Original Images for blots. (ZIP) [file pone.0297525.s004.zip › Original Images for Blots-2.tif]

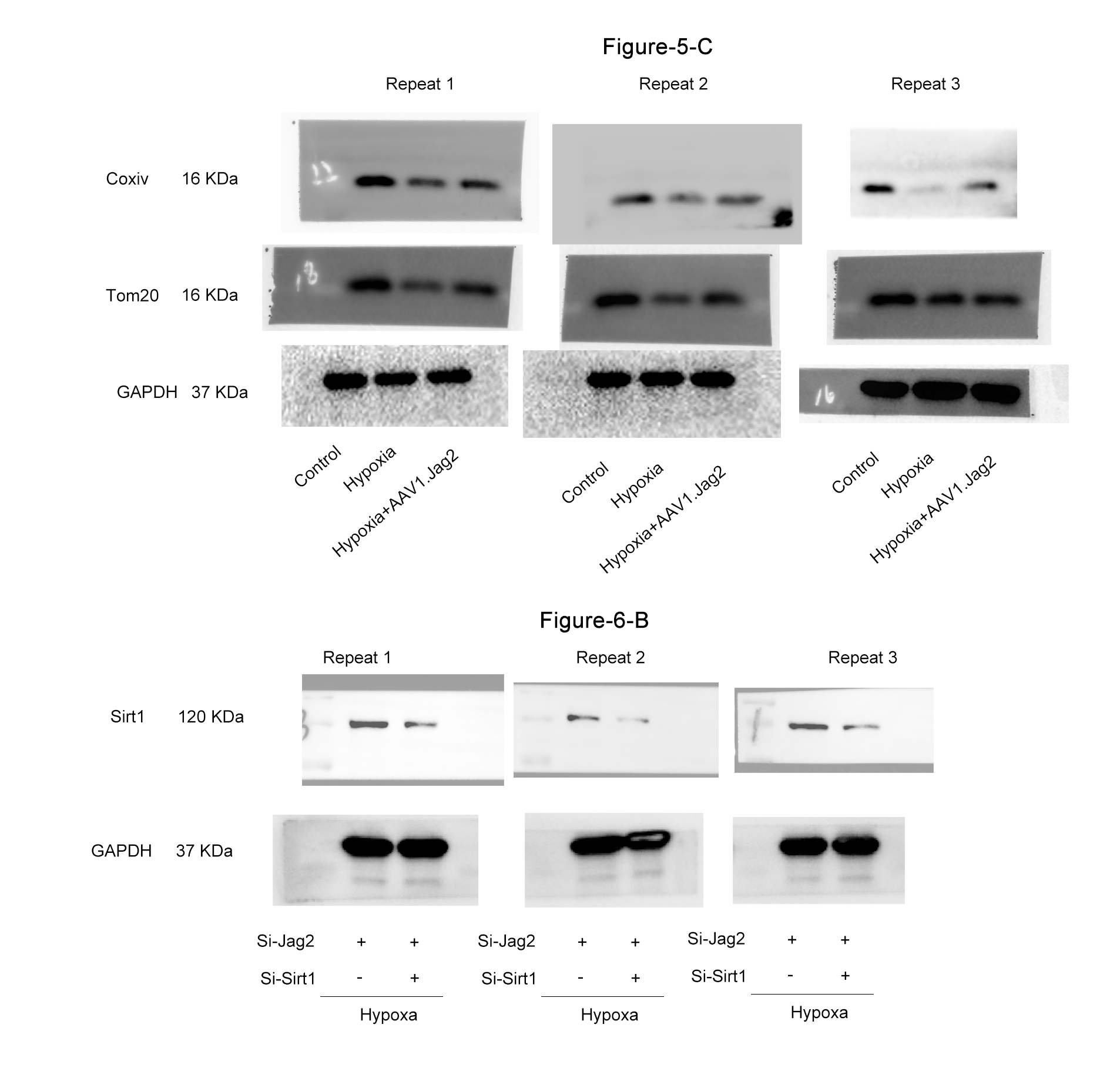

Supplement: S1 File — Original Images for blots. (ZIP) [file pone.0297525.s004.zip › Original Images for Blots-3.tif]

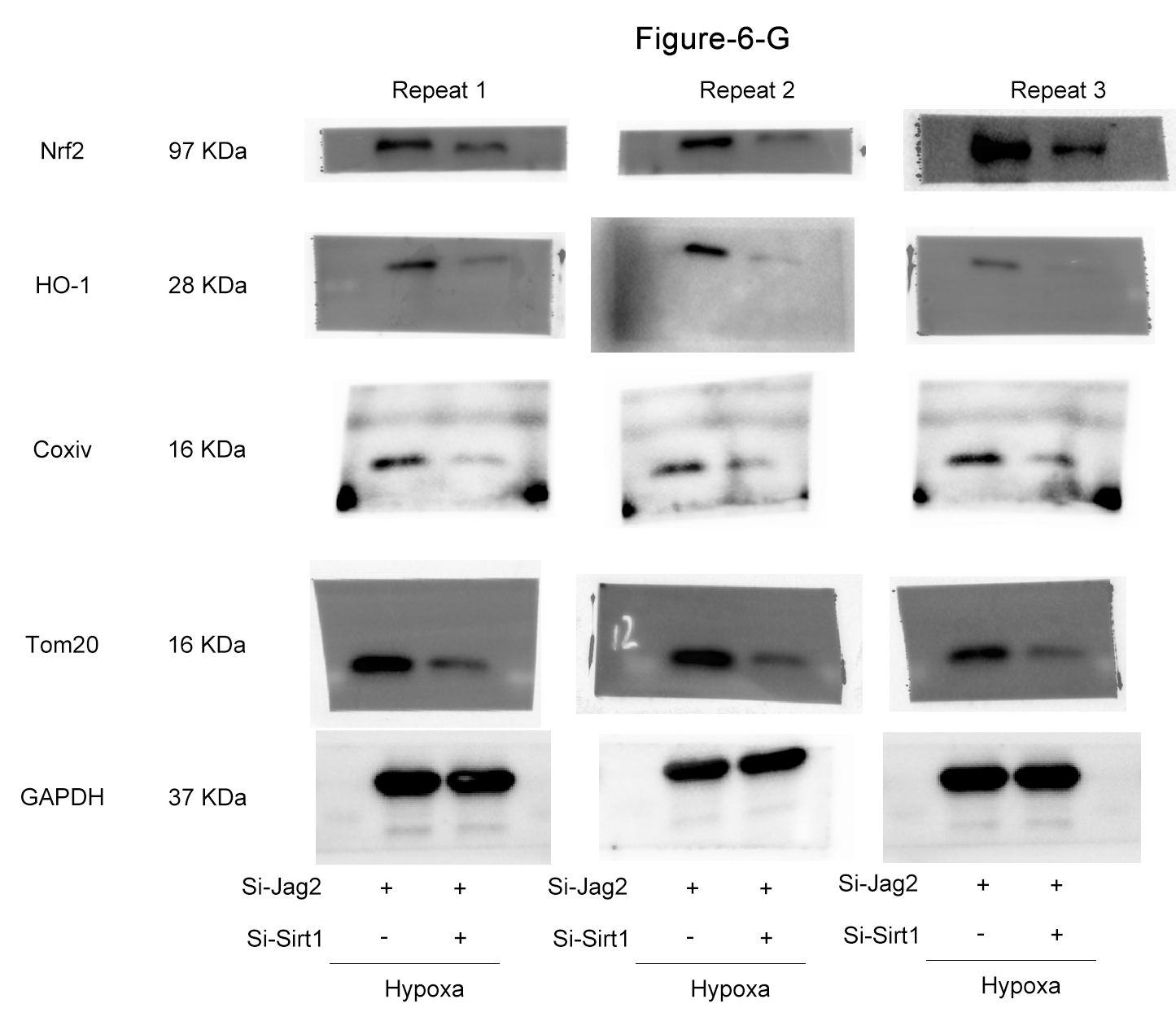

Supplement: S1 File — Original Images for blots. (ZIP) [file pone.0297525.s004.zip › Original Images for Blots-4.tif]

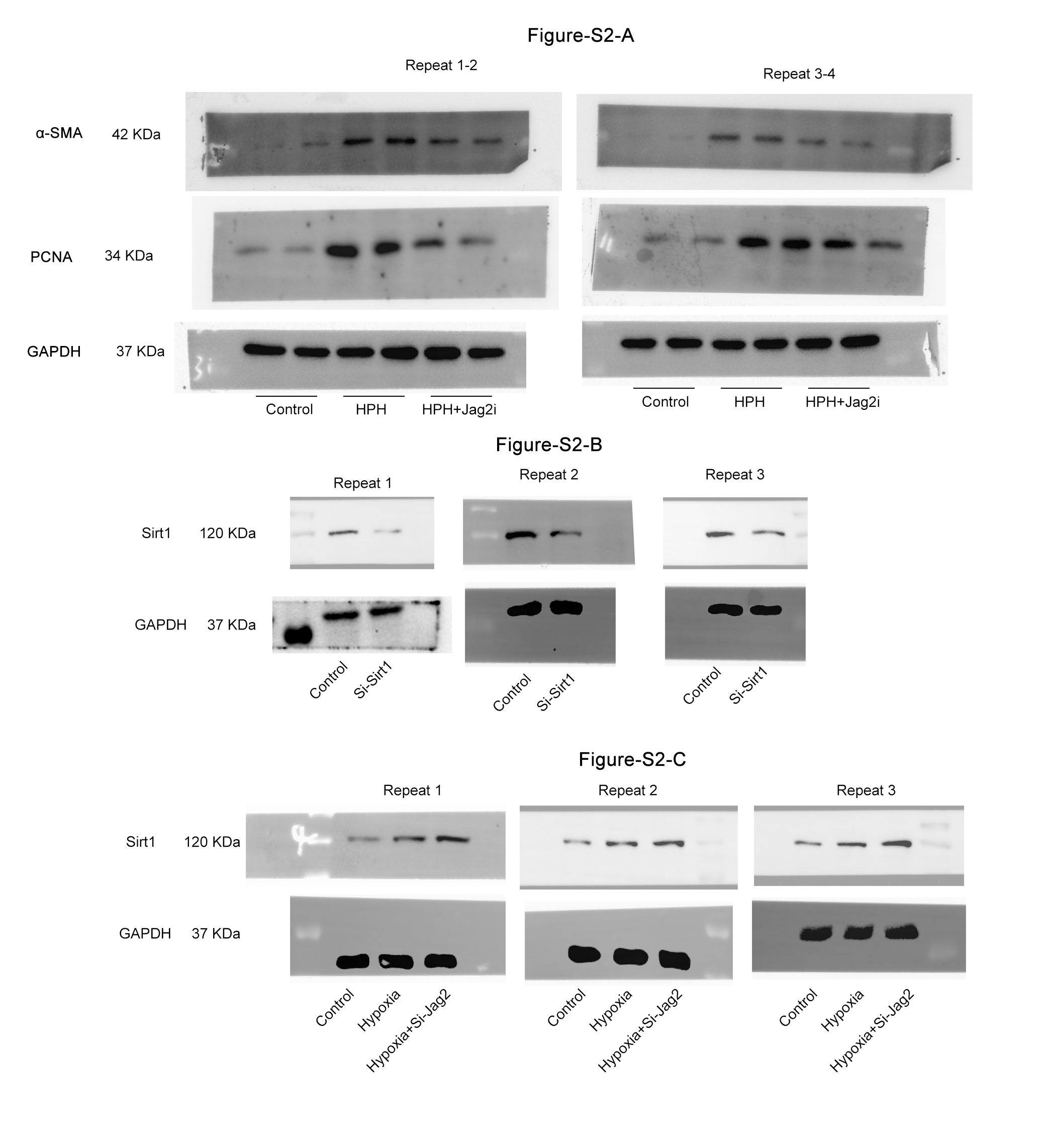

Supplement: S1 File — Original Images for blots. (ZIP) [file pone.0297525.s004.zip › Original Images for Blots-5.tif]

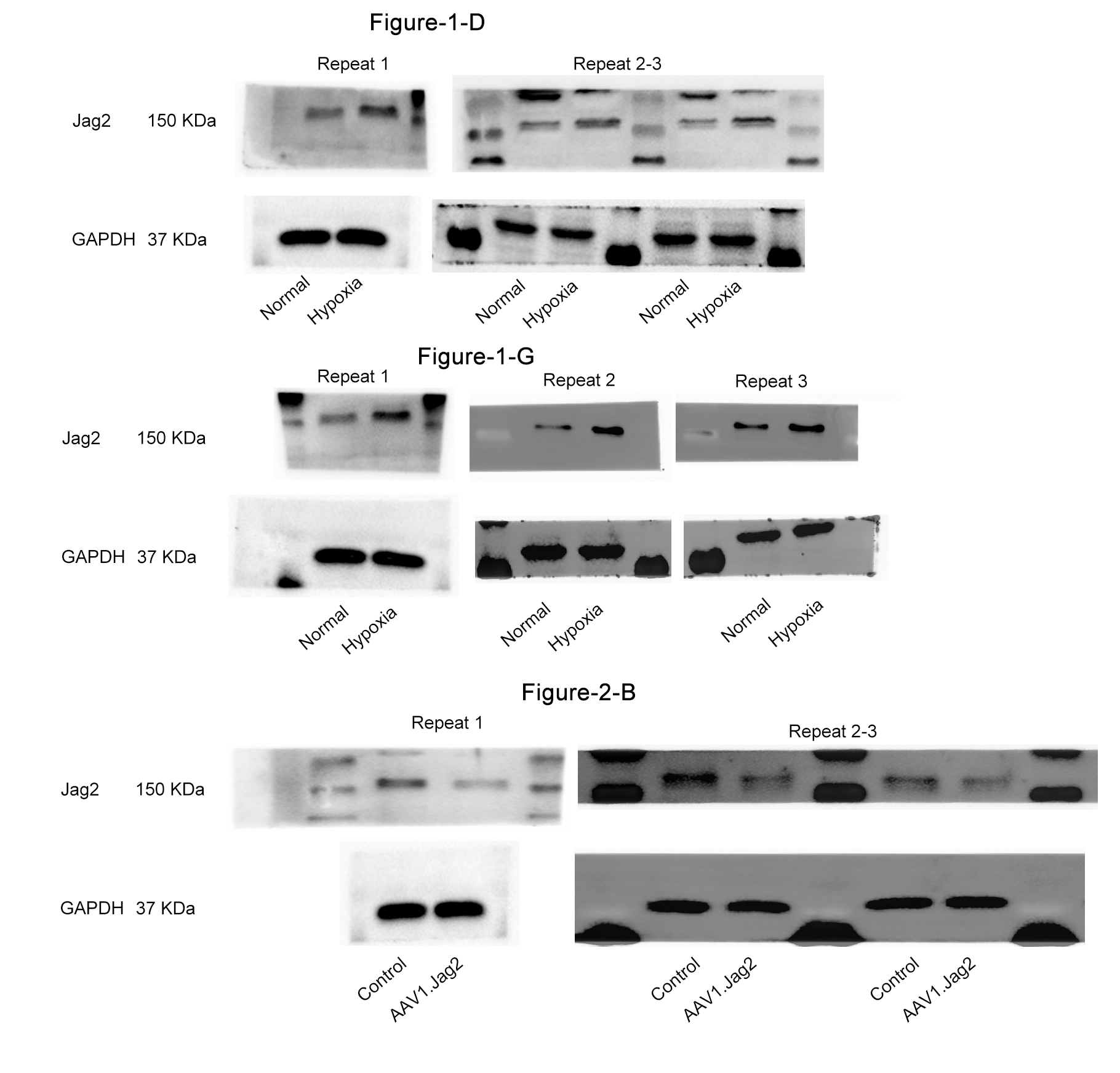

Supplement: S1 File — Original Images for blots. (ZIP) [file pone.0297525.s004.zip › Original Images for Blots-1.tif]
